# Early exercise improves cerebral blood flow through increased angiogenesis in experimental stroke rat model

**DOI:** 10.1186/1743-0003-10-43

**Published:** 2013-04-26

**Authors:** Pengyue Zhang, Huixian Yu, Naiyun Zhou, Jie Zhang, Yi Wu, Yuling Zhang, Yulong Bai, Jie Jia, Qi Zhang, Shan Tian, Junfa Wu, Yongshan Hu

**Affiliations:** 1 Department of Rehabilitation of Huashan Hospital, Fudan University, Shanghai, China; 2 Department of biomedical engineering, Shanghai JiaoTong University, Shanghai, China; 3 Department of Biomedical Engineering, Stony Brook University, New York, USA; 4 Genetic Diagnosis Center, Yunnan Provincial Key Laboratory For Birth Defects and Genetic Diseases, The First People's Hospital of Yunnan Province, Kunming, China; 5 The Yonghe Branch of Huashan Hospital, Fudan University, Shanghai, 200032, China

**Keywords:** Early exercise, Cerebral blood flow, Angiogenesis, Laser speckle flowmetry, Cerebral ischemia and reperfusion

## Abstract

**Background:**

Early exercise after stroke promoted angiogenesis and increased microvessles density. However, whether these newly formatted vessels indeed give rise to functional vascular and improve the cerebral blood flow (CBF) in impaired brain region is still unclear. The present study aimed to determine the effect of early exercise on angiogenesis and CBF in ischemic region.

**Methods:**

Adult male Sprague Dawley rats were subjected to 90 min middle cerebral artery occlusion(MCAO)and randomly divided into early exercise and non-exercised control group 24 h later. Two weeks later, CBF in ischemic region was determined by laser speckle flowmetry(LSF). Meantime, micro vessels density, the expression of tie-2, total Akt and phosphorylated Akt (p-Akt), and infarct volume were detected with immunohistochemistry, 2,3,5 triphenyltetrazolium chloride (TTC) staining and western blotting respectively. The function was evaluated by seven point’s method.

**Results:**

Our results showed that CBF, vessel density and expression of Tie-2, p-Akt in ischemic region were higher in early exercise group compared with those in non-exercise group. Consistent with these results, rats in early exercise group had a significantly reduced infarct volume and better functional outcomes than those in non-exercise group.

**Conclusions:**

Our results indicated that early exercise after MCAO improved the CBF in ischemic region, reduced infarct volume and promoted the functional outcomes, the underlying mechanism was correlated with angiogenesis in the ischemic cortex.

## Introduction

Ischemic stroke is a major cause of disability and death (American Heart Association, 2009[1]). With the advancement of the medical technology in the past decades, more and more stroke patients survived from the initial injury, however, most of them suffered from neurological deficits such as motor, study, memory and cognitive dysfunctions that reduce quality of daily life significantly [2,3]. The abruptly reduced focal blood flow is a predominant cause of stroke, which leads to the exhausted nutrients and oxygen. Subsequently, the injury cascade is initiated, such as release of excitatory glutamate, neuroinflammation, apoptosis, and edema etc. [4-7]. To salvage the affected cells, the rapid recovery of blood flow in the ischemic region was an effective treatment strategy in ischemic stroke [8-10]. However, the only thrombolysis agent supported by FDA, recombinant tissue plasminogen activator (tPA), was limited by its narrow therapeutic window and side effect [11]. Thus, it is necessary to find a strategy to supply impaired brain tissue with fully blood flow in delayed phase.

Angiogenesis is a process that forms new blood vessels. Although it occurs during normal embryonic development, some insults such as brain trauma and ischemia induced angiogenesis and vascular remodeling in adult [12,13]. Within a few hours after occlusion, hypoxia induced up-regulated expression of a group of angiogenic factors in infarct hemisphere including vascular endothelial growth factor (VEGF), Ang1/2 and their receptor Tie2. The synergistic effects triggered the proliferation of endothelial cells and vascular remodeling [14-16]. Using a brain vascular cast method, Krupinski et al. had described the vascular buds and connections in a rat model [17]. Similarly, immunohistological analysis and expression of mRNA studies had confirmed that angiogenesis was initiated within 48 hours after ischemia and persisted for up to a few weeks in rodent animal model [18-21]. New blood vessels increased the blood flow in affected region revealed by neuroimaging [22], it would be of important significance for improving the exchange of oxygen and glucose in hypoxic tissue, and subsequent functional outcomes [18,23,24]. Indeed, clinical study indicated that stroke patients with more newly formatted microvessels in the infarct region had survived a longer time [25,26]. Thus, increasing the angiogenesis after cerebral ischemia is a potential strategy for treatments of stroke.

Cumulative evidences indicated that exercise initiated soon after stroke (early exercise) protected against ischemia brain injury and improved functional recovery through angiogenesis, neurogenesis, suppressing apoptosis and neuroinflammation [27-32]. Evidences came from pre-clinical and clinic studies showed that exercise increased capillary density in motor cortex and improved CBF in normal condition [33-36]. These results implied that angiogenesis took a crucial role in post-stroke recovery. Indeed, in a mice model, Gertz et al. demonstrated that voluntary exercise after ischemia improved angiogenesis and CBF through eNOS-dependent mechanism and promoted the recovery of long-term outcomes [37]. Consisted with these findings, our recent results indicated that treadmill training initiated after stroke up-regulated the expression of angiopoietins and promoted the angiogenesis, reduced infarct volume and improved functional recovery in experimental stroke rats [30,38]. Though there is an increasing amount of evidences about the increased angiogenesis induced by exercise, it is not clear whether these newly formatted vessels indeed give rise to functional vascular in impaired brain.Laser speckle flowmetry (LSF) is a noninvasive imaging blood flow technique, which has widely been used to measure the CBF with high temporal and spatial resolution [39-42], and therefore, LSF can be allowed to examine the relative change of CBF at multi time points in same animal. Utilizing LSF technique in present study, we demonstrated that early exercise (2 weeks) after stroke improved angiogenesis in affected cortex; furthermore, rats with early exercise had an increased CBF, reduced infarct volume and promoted functional outcomes.

## Material and methods

### Rat middle cerebral artery occlusion (MCAO) model

All animal experiments were performed according to animal experimental committee of Fudan University at Shanghai, China. Adult male Sprague–Dawley rats (250-270 g, Shanghai SLAC Laboratory Animal Co. Ltd.) were housed under a 12:12 h light: dark cycle with food and water available ad libitum at 21 ± 1°C. Rats were anesthetized with 10% chloral hydrate (0.36 ml/kg i.p.); the left middle cerebral artery was occluded by the intraluminal suture technique described by Longa with some modified [43]. Briefly, a 4–0 nylon monofilament coated with a silicone tip was introduced from the carotid bifurcation into the internal carotid artery until mild resistance was felt. Reperfusion was established by gently withdrawing the filament after 90 min of occlusion. Free access to food and water was allowed after recovery from anesthesia. In the sham control group, all steps were included except for the occlusion of the middle cerebral artery.

### Treadmill training and group

**Figure 1:**
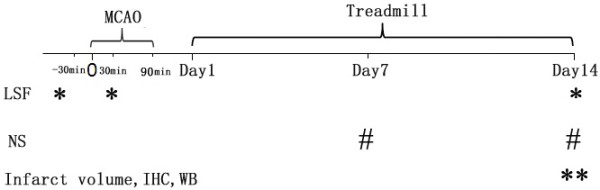
**Schematic illustration of the experimental design.** * represented the test points for laser speckle flowmetry (LSF). ** represented the test points for infarct volume, immunohistological staining (IH) and western blotting (WB). ^#^ represented the test points for neurological score (NS).

In order to reduce the stress of treadmill training, all rats were adapted to the treadmills (Litai Biotechnology Co., Ltd, China) at a speed of 6 m/min for 3 consecutive days (10 min per day) before MCAO. 24 hours after operation, all rats with MCAO were randomly assigned to early exercise and non-exercise group, the sham control was the third group, 18 rats were included in each group. Rats in early exercise group underwent treadmill training begun at 24 hours after MCAO for 14 consecutive days. The training intensity was gradually increased from 5 m/min at first day to 12 m/min at third day and persisted to 14^th^ day, which had been described in detail in our previous study [30]. The time points and duration were depicted in Figure 1 in detail. Rats in the remaining two groups were placed on the treadmill for 30 min without running [44].

### Laser speckle contrast imaging procedures and analysis

Anesthetized rat was fixed on a stereotaxic apparatus (David Kopf Instruments, Tujunga, California, USA). The scalp was incised and periosteum was removed. Then a 12×10 mm^2^ cranial window overlying the left cerebral cortex (1-5 mm lateral, between coronal suture and lambdoidal suture) was thinned evenly with high-speed dental drill (Fine Science Tools, North Vancouver, Canada) until the pial vasculature was visible. Cold saline was used to prevent damage caused by heating the surface of the brain during surgery. For imaging, rat was placed under a macro lens (Nikon 60 mm f/2.8 AF-S**,** Nikon Inc., Melville, New York, USA) in the stereotaxic apparatus. The aperture was adjusted to keep the speckle size comparable to the area of a single pixel in a 12-bit CCD (270XS 11066, Pixel fly, PCO, Kelheim, Germany) camera. The shutter speed was set to an exposure time of 5 ms and images were continuously taken at a rate of 23 fps. The stereotaxic could be moved along the x-y axes for location of the region of interest (full field within the cranial window). Finally, a 785 nm laser beam (L785P025, Visible Laser Diodes, Thorlabs China) was used to illuminate the cranial window in a diffuse and uniform manner. Before MCAO, the baseline LSF imaging has been conducted (100 consecutive frames of raw speckle images were recorded), and then 90 min transient focal cerebral ischemia was induced by left MCAO.30 min after occlusion, animal underwent a 15 min continuous LSF imaging monitoring (50 consecutive sets of 100 consecutive frames of raw speckle images were recorded). Two weeks later, all of the rats were taken another 15 min continuous imaging monitoring. These time points of test had been described in Figure 1.

**Figure 2:**
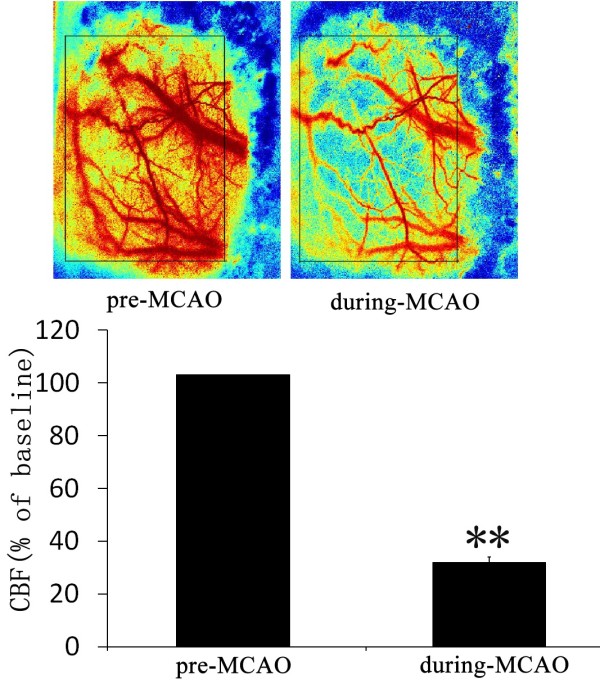
**MCAO induced a markedly ischemia in impaired cortex.** Above row there were the representative images of before and during MCAO, the bar graph showed the relative CBF (% of baseline).

**Figure 3:**
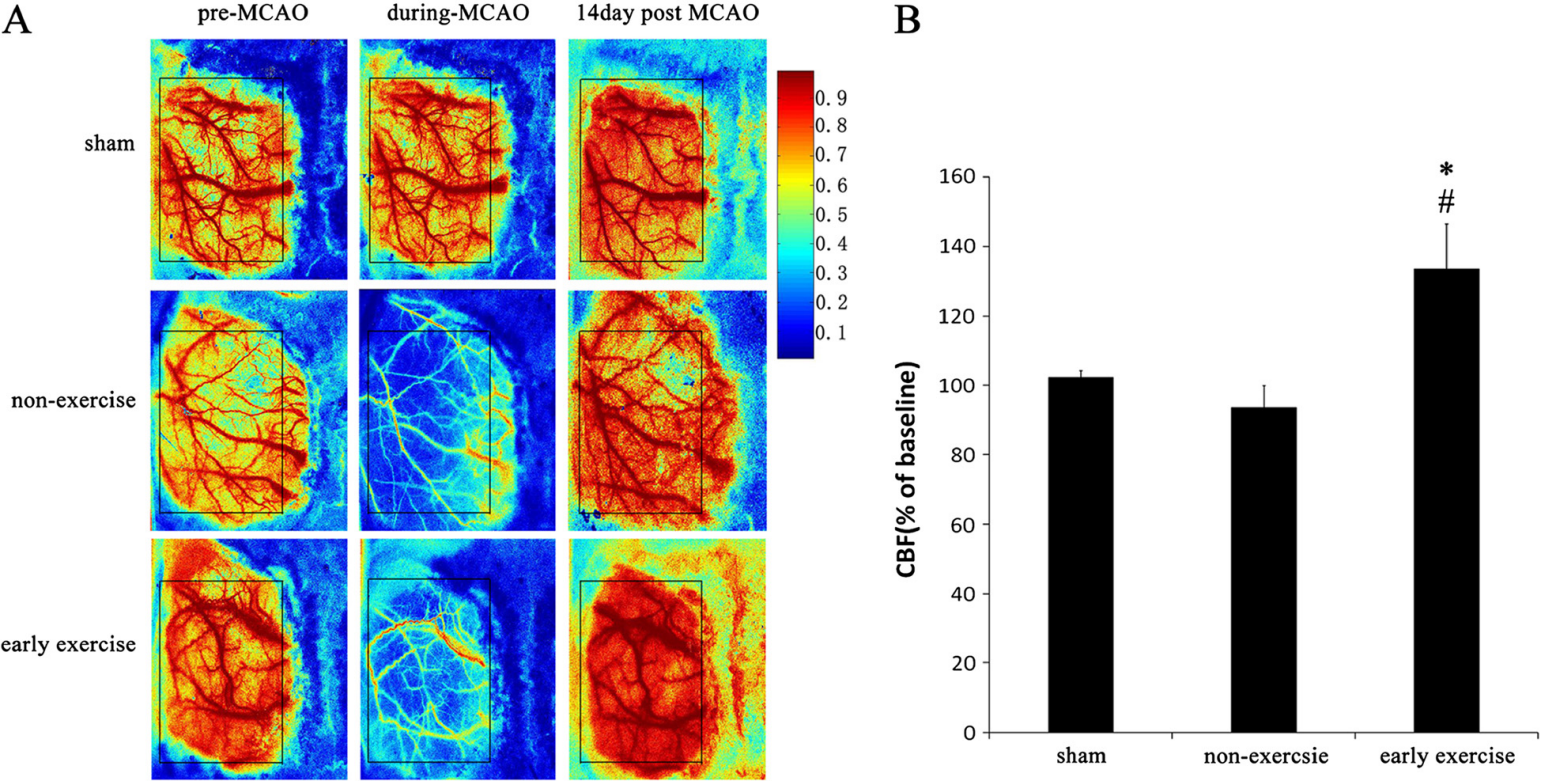
**Early exercise improved the CBF in ischemic cortex. A**, Representative CBF pseudocolor images in ischemia cortex before, during and 14 days post MCAO. **B**, Quantification of the relative CBF in ROI (% of baseline for each animal). n = 6 for each group. *p<0.05, versus the no-exercise group; #, p<0.05, versus the sham group. Color bar represents the capacity of CBF. Abbreviations: ROI, region of interesting.

Analysis of laser speckle images and relative CBF (rCBF) was performed using MATLAB 7.0 software (Mathworks, MA, USA). In order to increase signal-to-noise ratio, every 100 sequential raw speckle images were calculated to form one speckle contrast image. Because the infarct region has different among different animal, and exercise reduced the infarct volume in animal with exercise, we chose a region of interesting (ROI) including most supplied area by MCA as measured zone. The region of interesting was defined as a rectangular zone of 6 mm × 4 mm (2 mm lateral and 1mm anterior to the bregma) in each image, which had been described in Figures 2 and 3. In ROI, the 1/k^2^ were calculated according to published method [45,46], which was proportional to the velocity of red blood cells and on behalf of rCBF [40,42]. For every rat, the change of rCBF during MCAO and day-post-14 was defined as the ratio of the 1/k^2^ to its corresponding one of baseline. The ratios were used for statistical analysis.

### Determination of brain infarct volume

14 days after MCAO, 6 rats of each group were used to determinate brain infarct volume. Briefly, euthanasia was executed with 10% chloral hydrate, and brains were quickly removed and put in an ice-chilled rodent brain matrix (Braintree scientific, USA). Then the brain was cut into six consecutive coronal sections with 2 mm thickness. Then, these brain sections were rapidly put into 2% TTC (2, 3, 5-triphenyltetrazolium chloride) solution for 30 min at 37°C, followed by fixation in 4% paraformaldehyde buffer. The fixed sections were photographed with a digital camera (DC240; Kodak, USA). The pale area in section was defined as the infarct zone, which was traced and calculated using NIH Image software (available at: http://rsb.info.nih.gov/nih-image/). The percentage of infarct volume was determined according to an indirect method [47]: Infarct volume = (area of contralateral hemisphere − area of normal region in the ipsilateral hemisphere) / area of contralateral hemisphere× 100%. The results were presented as mean ± SE.

### Immunohistochemistry

14 days after MCAO, 6 rats of each group were anesthetized with 10% chloral hydrate and perfused with saline through the left cardiac ventricle followed by 4% paraformaldehyde (pH 7.4). Thereafter, brains were removed and dehydrated in 20% sucrose solution overnight. Frozen serial coronal brain sections were sliced on a cryostat (30 μm in thickness). For immunohistochemistry, Sections were blocked in 3% H_2_O_2_ and then in 10% normal goat serum (Jackson ImmunoResearch Laboratories, U.S.A.) for 1 hour respectively. After that, sections were incubated with rabbit anti-rat CD31 (Abcam, Cambridge, MA, 1:200) overnight at 4°C. At the next day, sections were stained with biotinylated goat anti-rabbit IgG secondary antibody (KPL, 1:200) at 37°C for 1 hour, followed by preformed avidin-horseradish peroxidase complex (Vectastain Elite ABC-Reagent, Vector) for another 30 min. Diaminobenzidine (Sigma-Aldrich) was used for immunostaining and hematoxylin was used for counterstaining nuclei. The sections were finally dehydrated and clarified through a graded series of ethanol and xylene, then mounted under coverslips using neutral gum. The number of positive cells was counted in penumbra of hemisphere with the lesion under the light microscope (400×). For each section, five visual fields in penumbra of hemisphere with the lesion were chosen at random for statistical analysis. Results were expressed as the mean number of CD31 positive cells per mm^2^.

### Protein isolation and Western blotting

Cortex tissues of the ischemic hemisphere were homogenized in RIPA Lysis buffer (Beyotime Biotechnology, China) and clarified by centrifugation (14000 g, 20 min, and 4°C). Supernatants were harvested and protein concentrations were measured using the bicinchoninic acid assay (BCA; Beyotime Biotechnology, China). For gel electrophoresis, samples were separated on 12% SDS-polyacrylamide gels, and then transferred onto polyvinylidene fluoride (PVDF) membranes (Millipore, USA). Membranes were blocked for 1h with 5% w/v bovine serum albumin (Roche, USA) at room temperature followed by incubated overnight with primary antibody against Tie2 (Abcam, Cambridge, MA, 1 μg/ml) ,Akt and Phospho-Akt (Ser473) (Cell signaling technology, Massachusetts, U.S.A., 1:1000) at 4°C. After three washes, membranes were incubated for 1 h at room temperature with horseradish peroxidase (HRP)-conjugated antirabbit IgG (Jackson, U.S.A, 1:2000). Detection was performed by pierce ECL kit (Thermo Scientific, U.S.A). Bands were quantified by fluorescence densitometry using a commercial imaging System (Bio-Rad, U.S.A). Western blotting signals were normalized against the signals obtained with horseradish peroxidase (HRP)-conjugated mouse monoclonal anti-glyceraldehyde-3-phosphate dehydrogenase (GAPDH; Kandchen, China).

### Neurological deficits scores

At 1st, 7th and 14th day after MCAO, neurological deficits scores were tested as previously described [30,48], all rats were scored by an observer blinded to experiment design with the following criteria: 0, no neurological symptoms; 1, unable to extend right forepaw fully; 2, reduced grip of the right forelimb; 3, torso turning to the right side when held by tail; 4, circling or walking to the right; 5, failure to walk without help; 6, no spontaneous activity or narcosis; and 7, dead.

### Statistical analysis

Data are presented as means ±standard error of the mean (SEM). Statistical differences were assessed by one-way analysis of variance (ANOVA) followed by post hoc Fisher’s PLSD tests. P<0.05 was considered statistically significant.

## Results

### Change of cerebral blood flow during MCAO

The change of CBF before and after operation were determined in all of rats by LSF, the results showed that MCAO induced a significant CBF decline (31.07%±1.95% of baseline, Figure 2). In contrast, there was no change of CBF in sham group after operation.

### Early exercise improved the CBF in ischemic region

MCAO rapidly damaged the cerebral microvessel and led to a hypoperfusion in ischemic cortex and striatum, simultaneously, an emergency response system was started to compensate the depressed CBF. These process included construction of collateral circulation and angiogenesis in impaired brain tissue. Two weeks after MCAO, we observed that the CBF returned to baseline level (Figure 3A). Compared with non-exercise group, the early exercise furthermore improved the recovery of CBF that reached to 1.3 times of baselines (Figure 3). In contrast, the CBF in sham group was not different from baseline at the 14^th^ day. Our results suggested that early exercise improved the recovery of CBF in ischemic cortex.

### Early exercise promoted the angiogenesis in ischemic region

**Figure 4:**
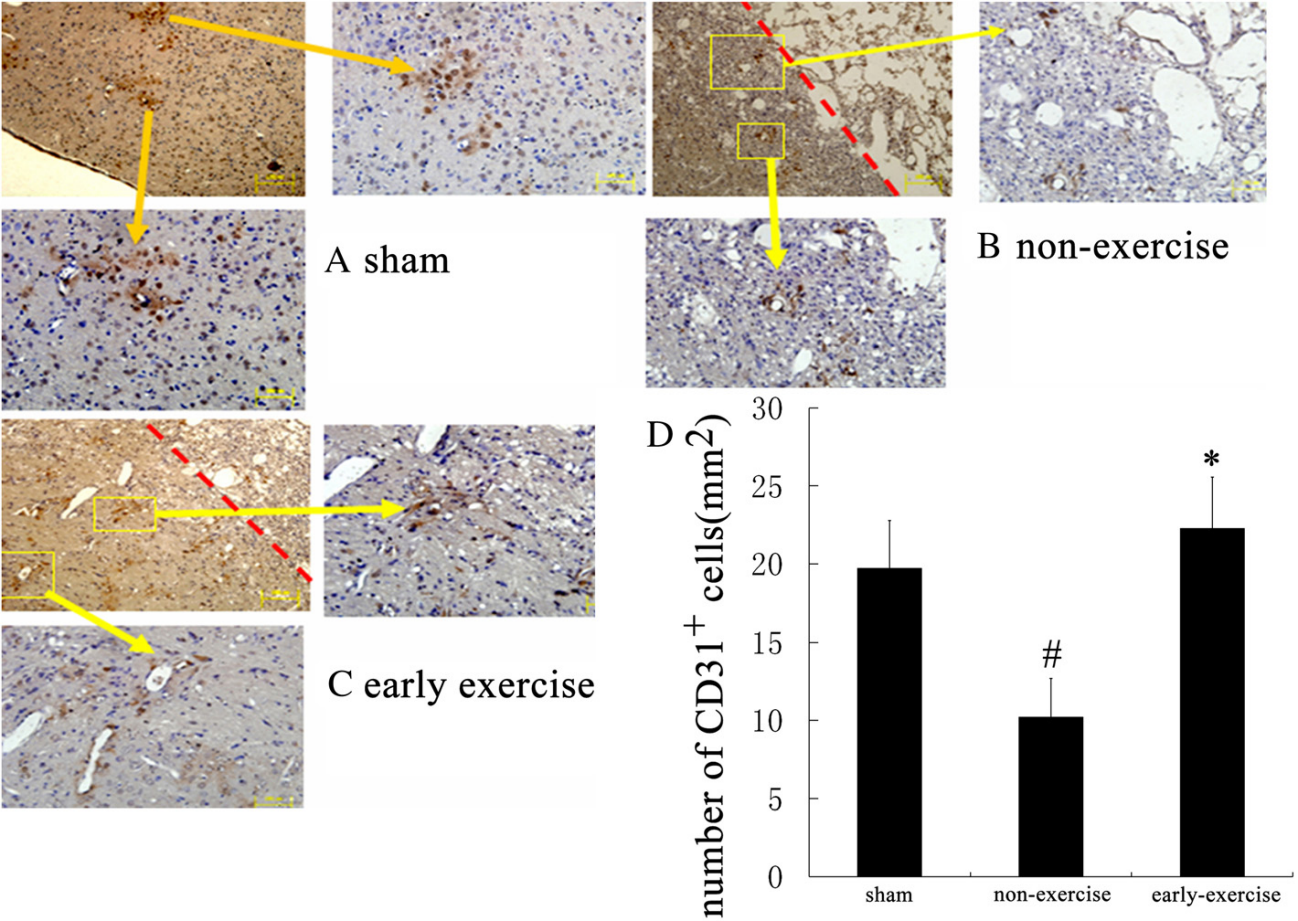
**Early exercise promoted angiogenesis in ischemic cortex.** In the early exercise group, intensive blood vessels (CD31^+^endothelial cells) were found in perilesional zone (**C**). In contrast, in non-exercised group (**B**), the density of micro vessels was much lower than the early exercise (**C**) and sham groups (**A**). The red dotted line indicated the infarct zone in upper left corner. (**D**) Quantification of the CD31^+^ cells showed that there was a significantly greater micro vessels density in the early exercise group. n = 6 for each group. *p<0.05, versus the no-exercise group; #, p<0.05, versus the sham group.

Immunocytochemistry was used to label brain microvessel endothelial cells. In all groups, many blood vessels were intensively marked by CD31 monoclonal antibody around the ischemic region (Figure 4). In the non-exercise group (Figure 4B), the number of positive cells was sparse compared to the early exercise group (Figure 4C) and the sham group (Figure 4A) in penumbra. Quantitative analysis showed that density of micro vessels in the early exercise group was significantly higher than non-exercise group (22.3±3.3 per mm^2^ in early exercise group vs. 10.2±2.5 per mm^2^ in non-exercise group, p<0.05), and the number of CD31 positive cell in early exercise group was no different from that in sham group (19.7±3.1 per mm^2^). These data indicated that two weeks exercise promoted the proliferation of microvessel endothelial cells and angiogenesis.

### Early exercise enhanced the expression of angiopoietins

**Figure 5:**
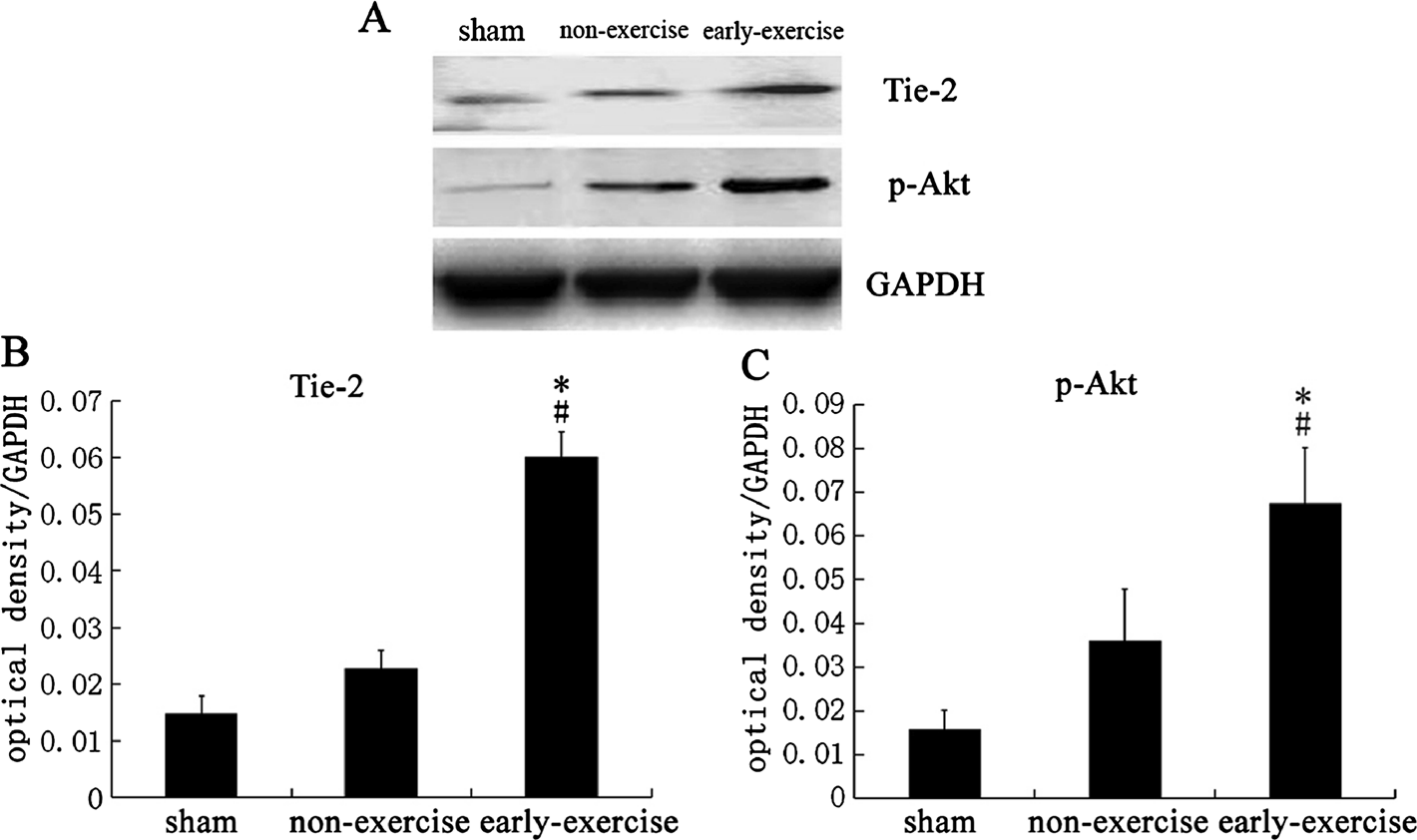
**Early exercise increased expression of Tie-2 and p-AKT.A.** Representative images of Western Blotting for Tie-2, p-AKT and GAPDH. **B** and **C**, Quantification of the optical density for Tie-2 and p-AKT, normalized to GAPDH. There was a significantly increased expression of Tie-2 and p-AKT in early exercise group. n = 6 for each group. *p<0.05, versus the no-exercise group; #, p<0.05, versus the sham group.

Tie-2 is a critical receptor of angiopoietins who take part in angiogenesis after MCAO. Our results showed that the expression of Tie-2 in early exercise group was significantly greater than the other two groups (Figure 5). There was no significant difference between the sham group and non-exercise group. Furthermore, we detected the expression of total Akt and p-Akt, an important protein which involved in cell survival and proliferation. We found that early exercise increased the expression of p-Akt significantly compared to the other two groups (Figure 5). However, the exercise didn’t affect the expression of total Akt (data not show) that consisted with the results from post-conditioning’s protection against stroke [49]. These data showed that the expression of tie-2 receptor, angiopoietin and p-Akt was increased significantly in early excise group.

### Early exercise reduced the infarct volume

**Figure 6:**
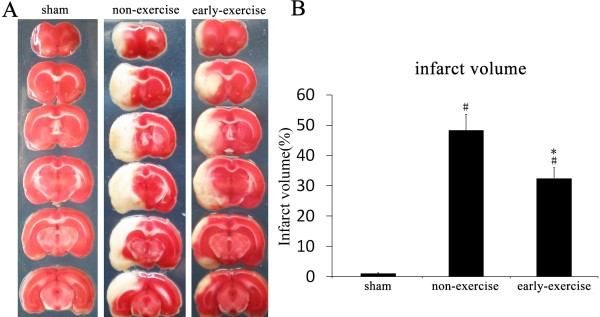
**Early exercise reduced infarct volume induced by MCAO. A**, Representative images of TTC stained section. **B**, Quantitation of the infarct volume showed that early exercise significantly reduced infarct volume after MCAO compared to non-exercise rats. n = 6 for each group. *p<0.05, versus the no-exercise group; #, p<0.05, versus the sham group.

The cerebral infarct volume was measured at 14th day after MCAO (Figure 6). Compared to the non-exercise group, early exercise significantly reduced the infarct volume (48.35±6.03% vs. 32.46±3.81% in non-exercise and early exercise groups, respectively, p<0.05) (Figure 6), and the rats in the sham group did not exhibit any infarct region (Figure 6). The results showed that early exercise after MCAO was able to reduce infarct volume significantly.

### Early exercise improved functional outcomes

**Figure 7:**
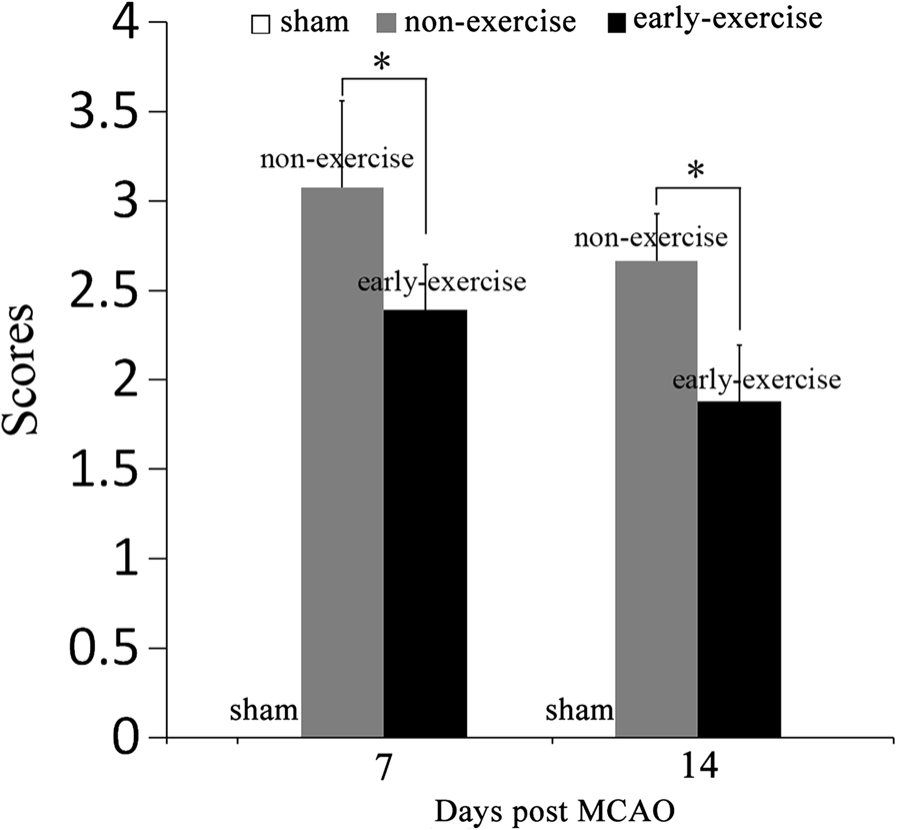
**Early exercise improved neurobehavioral recovery.** MCAO caused a markedly neurological deficit, and early exercise reduced the neurological score at 7 day and 14 day after MCAO compared to non-exercise group. In contrast, rats in sham group performed no neurological deficit. n = 12 for each group. *p<0.05, versus the no-exercise group.

All rats with MCAO exhibited severe neurological deficits at first day (before the early exercise), and there was no statistic difference between early exercise group and non-exercise group (4.06±0.86 vs. 4.12±1.02 in non-exercise and early exercise groups, respectively). The effect of early exercise on recovery of function was evaluated at 7th and 14th day after MCAO. We observed that early exercise significantly promoted functional outcomes at 7th and 14th day after MCAO (p<0.05, Figure 7). All rats in sham group exhibited no neurological deficits at 1st, 7th and 14th day after MCAO. The results indicated that early exercise improved the recovery of function after MCAO.

## Discussion

Increasing researches in both stroke patients and ischemic animal models had indicated that exercise initiated after MCAO protected against cerebral ischemia and improved functional recovery [31,32]. In our recent study, we found that early exercise inhibited acute neuroinflammatory response and promoted the functional recovery from MCAO [30]. Here, using the LSF technology we demonstrated that two weeks treadmill training after stroke improved CBF, reduced brain infarct volume, and promoted functional recovery in an experimental stroke rat model, the possible mechanism involved in angiogenesis in ischemic cortex.

Within minutes of MCAO, the core of the brain tissue that exposed to the most dramatic blood flow reduction is fatally injured and subsequently undergoes necrotic cell death. This necrotic core is surrounded by a zone of less severely affected tissue that is rendered functionally silent by reduced blood flow, but these cells in this area remain metabolically active. This border region, known as the “ischemic penumbra”, may comprise as much as half of the total lesion volume during the initial stages of ischemia, thus there is an opportunity for salvage via post-stroke therapy in this region [50]. The rapid recovery of CBF to normal level in ischemic hemisphere is very important for rescue of the cells in penumbra and the functional recovery. Thrombolytic reagent used in clinic currently can restore CBF in ischemia area and facilitate the functional recovery, but was limited by its narrow therapeutic window and side effect [11].

Angiogenesis was a process involving in proliferating and sprouting of endothelial cells and subsequent formation of new vessels, which played a critical role in functional recovery of brain insults including stroke and TBI [13,51]. Ischemic stroke damaged the vessels net in impaired tissue, on the other hand, stroke stimulated angiogenesis that aimed to constitute the new vessels net and rescue the neuron in ischemic penumbra [52,53]. Even in the first week, the increased microvessel density had been observed [12,18]. Utilizing serial magnetic resonance imaging, Lin and colleagues [54] observed an increased cerebral blood volume in ischemic hemisphere from 3 to 21 day after experimental stroke. In present study we detected the CBF in the region supplied by MCA, including infarct core and penumbra in ischemia hemisphere, and we found that the CBF in ischemic cortex had reached to the baseline level after two weeks spontaneous recovery.

Reports from healthy human and animal suggested that exercise increased the expression of angiogenic growth factor including VEGF, VEGF receptor and angiopoietin receptor, and improved the blood flow capacity in skeletal muscle [55-57] and brain tissue [58]. When initiated after cerebral ischemia, exercise increased the expression of angiopoietins and their receptor in ischemia region, such as Ang2/Tie-2 and VEGF/VEGFR. Ang-2 is the most prominent member of a family of angiogenic growth factors, which promotes angiogenesis through its receptor Tie-2, a receptor of tyrosine kinases that play essential roles in angiogenesis [59,60]. In stroke rat, Ang2/Tie2 was up-regulated during the first 24 hour and lasted up to a few weeks after MCAO [61,62]. Increased expression of Ang2/Tie-2 stimulated the sprouting of endothelial cells and development of new vessels, and then enhanced the microvessels density [21,63]. In our previous study, we observed that 2 weeks treadmill training up-regulated Ang-1 mRNA expression in the ischemic cortex [38]. Here, we furthermore conformed that early exercise increased Tie2 expression by western blotting, which consisted with the enhanced microvessels and improved CBF in ischemia cortex.

In addition to pro-angiogenic factor, the proliferation of endothelial cells is another important aspect that supports angiogenesis and formation of new vessels. Akt is a critical factor for endothelial cell survival and proliferation in cerebral ischemia injury [64-66]. In the present study we determined the expression of total and phosphorylated Akt (p-Akt). Our results indicated that two weeks treadmill training increased the expression of p-Akt but not total Akt which were consisted with the results from postconditioning’s protection against stroke [49]. In order to examine the enhanced angiogenesis by exercise, we detect the density of microvessel endothelial cells in ischemia region; and our results show that early exercise increased the density of microvessle endothelial cells significantly. These results were consisted with the improved CBF and functional outcomes.

Enhanced angiogenesis not only increased CBF in ischemia region, but also stimulated the neurogenesis, both of them facilitated the functional recovery. Using condition medium, Teng et al. [67] demonstrated that endothelial cells from ischemic brain tissue stimulated neural stem cells proliferation and neuronal differentiation in vitro. The underlying mechanisms involved the pro-angiogenesis factor VEGF and chemokine stromal derived factor 1α (SDF-1α) secreted from endothelial cells [67-70]. Administration or over expression VEGF increased neurogenesis after stroke in pre-clinic study [14,71]. SDF-1α was an important chemokine which mediated neuroblast migration along the cerebral vessels, and blockade of this pathway abolished stroke-induced neuroblast migration [72-74]. Combining the MRI approach, Pereira and coworkers demonstrated that improved cerebral blood volume induced by exercise was correlated with enhanced hippocampus neurogenesis in mouse and promoted cognitive function in human [75]. In our previous study we had observed the promoted learning and memory ability after two weeks treadmill training post MCAO [30]. Here, we detected the increased angiogenesis and improved CBF in early exercise group, which were correlated with the better functional outcomes. These results implied that improved CBF induced by early exercise in ischemia cortex promoted the functional recovery.

By detecting the speckle contrast values, which is inversely related to blood flow velocity, LSF can monitor real-time dynamic of CBF changes in the same animal at multiple time points. This technology make it possible to compare CBF changes between pre and post treat of stroke, which is useful to assess the effect of therapeutic intervention. However, because of the limited penetrating ability of laser, LSF only detects the CBF in surface layer of cortex (about 1 millimeter in depth). So the enhanced CBF in our results was only detected in the out layer of cortex. The CBF changes in deeper brain tissue needed to be elucidated in future. Another limitation of present study was the lack of a group with sham and exercise. So we couldn’t compare the CBF and angiogenesis between the normal and the ischemic condition. Despite of these, the present data revealed that early exercise markedly induced angiogenesis, improved the CBF in ischemic region, and promoted functional outcomes after MCAO, and this work provided a support for clinical application of rehabilitation at the early stage of cerebral ischemia.

## Conclusions

In this study, we showed that early exercise after MCAO increased density of microvessels and improved blood flow capacity in the ischemic cortex, reduced infarct volume and promoted the functional outcome. These results implied that the newly formatted vessels were functional and the angiogenesis may be one of the important mechanisms in functional recovery.

## Abbreviations

CBF: Cerebral blood flow; LSF: Laser speckle flowmetry; MCAO: Middle cerebral artery occlusion.

## Competing interests

There is no competing interest.

## Authors’ contributions

PZ participated in the experiment design, carried out the determination of cerebral blood flow, analysed infarct volume and interpreted of these data, prepared the manuscript. HY participated in MCAO, carried out the western blotting detect and the immunohistological staining, NZ performed the determination of cerebral blood flow and analysis of the results, helped to draft the manuscript. JZ performed neurologic deficits score and statistical analysis, YW and YZ participated in the design of the study and performed the statistical analysis, YB participated in experiment design and coordination, helped to draft the manuscript. JJ and JW participated in MCAO and interpretation of data, QZ and ST performed neurological score and analyzed the results, and participated in MCAO. YH conceived and designed the experiments, interpreted the results and wrote the manuscript. All authors read and approved the final manuscript.
